# Ensemble learning for predicting microsatellite instability in colorectal cancer using pretreatment colonoscopy images and clinical data

**DOI:** 10.3389/fonc.2025.1734076

**Published:** 2026-01-02

**Authors:** Jia You, Shenghan Zhang, Jianjie Zhang, Yaru Chen, Mengmeng Zhang, Chungen Zhou, Bin Jiang

**Affiliations:** 1 Nanjing Hospital of Chinese Medicine Affiliated to Nanjing University of Chinese Medicine, Nanjing, Jiangsu, China; 2 Department of Biomedical Informatics, Harvard Medical School, Boston, MA, United States

**Keywords:** artificial intelligence, colonoscopy, colorectal cancer, deep learning, diagnositic model, ensemble learning, machine learning, microsatellite instability (MSI)

## Abstract

**Background:**

Microsatellite instability (MSI) is an important molecular biomarker in colorectal cancer (CRC), associated with favorable prognosis and response to immune checkpoint inhibitors. Conventional MSI testing, including immunohistochemistry (IHC) and polymerase chain reaction (PCR), is invasive, time-consuming, and resource-dependent, underscoring the need for non-invasive and automated alternatives. This study aimed to develop and evaluate an ensemble learning framework integrating pretreatment colonoscopy images and routine clinical data for non-invasive MSI prediction in CRC.

**Methods:**

In this retrospective study, patients with pathologically confirmed CRC and IHC-determined MSI status were included. Pretreatment colonoscopy images and routine clinical variables were collected. Five deep learning architectures (ResNet-50, EfficientNet, DenseNet, VGG-16, and Vision Transformer) were trained on image data, while four machine learning algorithms (Logistic Regression, Random Forest, Support Vector Machine, and Gradient Boosting) were trained on clinical data. The best-performing models from each modality were combined using a majority-voting ensemble. Model performance was assessed using accuracy, precision, recall, and area under the receiver operating characteristic curve (AUROC). Interpretability was evaluated using Gradient-weighted Class Activation Mapping (Grad-CAM) for image models and SHapley Additive exPlanations (SHAP) for clinical models.

**Results:**

Among 1,844 patients, VGG-16 achieved the best image-based performance (AUROC = 0.896, accuracy = 0.832, recall = 0.708). Logistic Regression outperformed other clinical models (AUROC = 0.898, accuracy = 0.825, recall = 0.828). The ensemble model integrating both modalities achieved AUROC = 0.886, precision = 0.920, and recall = 0.845, outperforming single-modality approaches.

**Conclusion:**

The proposed ensemble learning framework provides a non-invasive, interpretable, and accurate method for MSI prediction, offering potential to improve preoperative precision diagnostics and clinical decision-making in colorectal cancer.

## 1 Introduction

Colorectal cancer (CRC) is the third most commonly diagnosed cancer and the second leading cause of cancer-related mortality worldwide, accounting for more than 900,000 deaths annually (1). Microsatellite instability (MSI), resulting from deficiency of the mismatch repair (MMR) system, is a key molecular subtype of CRC with critical clinical implications (2). MSI is associated with a more favorable prognosis in early-stage disease, particularly in stage II CRC (3). In addition, MSI tumors display marked responsiveness to immune checkpoint inhibitors, largely attributable to their high mutational burden and immunogenic microenvironment, making MSI status a crucial biomarker for guiding immunotherapy (4). Moreover, MSI serves as the molecular hallmark of Lynch syndrome, the most common hereditary colorectal cancer syndrome, and its detection is essential for identifying affected patients as well as at-risk family members (5). Consequently, MSI testing has become indispensable for guiding therapeutic decisions, predicting prognosis, and Lynch syndrome screening.

Current MSI testing primarily relies on immunohistochemistry (IHC) for MMR proteins and polymerase chain reaction (PCR)-based assays. Both approaches require tissue samples obtained through colonoscopy biopsy or surgical resection, which are inherently invasive and may lead to complications such as infection or bleeding. Tumor heterogeneity may also result in sampling bias, with MSI status underestimated or overestimated depending on the biopsy site (6). Furthermore, conventional testing methods often require several days to generate results and depend on specialized laboratory infrastructure, trained pathologists, and quality-controlled reagents, which are not universally available, particularly in resource-limited settings (7). These limitations underscore the urgent need for non-invasive, real-time, and cost-effective alternatives to support precision oncology.

Artificial intelligence (AI) has achieved rapid progress in medical image analysis (8, 9), thereby providing promising opportunities for MSI prediction. Pathology-based models applying deep learning to hematoxylin and eosin (H&E) slides have achieved high predictive accuracy by identifying morphological patterns associated with MSI (10, 11). Likewise, radiology-based approaches using CT and MRI have shown promise, either through end-to-end deep learning models applied directly to imaging data (12) or through radiomics workflows in which high-dimensional quantitative features are extracted and subsequently modeled using machine learning algorithms (13–15). However, pathology-based methods remain invasive, and radiology-based approaches often require labor-intensive manual tumor segmentation and rely on imaging features that may not fully capture biologically relevant tissue characteristics.

Colonoscopy offers a compelling alternative. It is routinely performed for CRC screening, localization, and treatment (16, 17), enabling the acquisition of high-quality, pretreatment images that reflect mucosal morphology, vascular patterns, and surface texture in real time. Compared with radiology, colonoscopy provides richer visual information at lower cost and without radiation exposure. Recent studies have demonstrated the feasibility of using colonoscopy images for MSI prediction (18, 19). For instance, Lo et al. (19) developed a Vision Transformer (ViT) model that achieved an AUC of 0.86 in MSI detection, while Cai et al. (18) trained a convolutional model achieving AUROCs of 0.948 (internal) and 0.807 (external). Despite these advances, most image-based approaches remain unimodal, relying solely on visual information, which may limit their robustness and interpretability.

Recent evidence suggests that integrating multiple data modalities can significantly enhance model performance. Multimodal AI frameworks have shown an average AUC improvement of approximately six percentage points over unimodal models across medical domains (20). For MSI prediction, combining clinical features with pathology (21) or radiology data (13, 14, 22) has been shown to improve accuracy. Within colonoscopy, Lo et al. (23) proposed a multimodal ViT model that concatenated colonoscopy image features with clinical data to predict colorectal cancer prognosis, achieving an AUC of 0.93 compared with 0.77 for colonoscopy images alone and 0.59 for clinical features alone. However, direct feature concatenation between heterogeneous data types may not optimally capture cross-modal relationships, emphasizing the need for more effective integration strategies.

Ensemble learning offers a practical and generalizable solution by combining outputs from multiple models to improve predictive stability and generalization. For instance, Cui et al. (24) applied a multimodal AI framework to the diagnosis of solid pancreatic lesions by integrating an endoscopic ultrasound imaging model with a clinical data model, achieving superior diagnostic accuracy compared with unimodal approaches. Likewise, recent work in bone tumor classification demonstrated that combining radiological imaging with clinical data outperformed image-only strategies (25). Building on these findings, integrating colonoscopy images with clinical data via ensemble learning may provide a non-invasive, interpretable, and clinically scalable approach for MSI prediction in CRC.

In this work, we developed and evaluated five deep learning architectures for colonoscopy image analysis and four machine learning classifiers for clinical data. Based on performance and balance across evaluation metrics, we selected representative models from each modality and integrated them using a majority-voting strategy to construct a multimodal ensemble framework. To enhance interpretability and facilitate clinical translation, we further applied Grad-CAM to visualize model attention in image-based predictions and SHAP to identify key feature contributions in the clinical models.

## 2 Materials and methods

### 2.1 Study design

We retrospectively identified patients with CRC treated at Nanjing Hospital of Chinese Medicine between 2019 and 2024. Eligible patients met the following inclusion criteria: (1) Pathologically confirmed CRC (2); Available MSI status determined by IHC. Patients were excluded if they had (1) received radiotherapy, chemotherapy, immunotherapy, or surgical resection prior to MSI testing or (2) synchronous colorectal tumors. MSI status was defined as loss of expression of at least one MMR protein, while preserved expression of all four proteins was classified as MSS.

Colonoscopy images were collected from the hospital’s picture archiving and communication system (PACS). These images were obtained from patients undergoing colonoscopy for cancer screening and tumor localization. Images were obtained using multiple endoscopy platforms, including Olympus Medical Systems (CF-H260AI, CF-H290I, CF-Q260AL, CF-H170I), Fujifilm Medical Systems (EC-530WI, EC-L590ZW, EC-530WM, EC-600WM, EC-760R-V/M, EC-760ZP-V/M), and Pentax Medical Systems (EC-34-i10F, EC-38i10F, EC-3890Fi, EC-3890FK, EC-3870FK). All images were exported in their original resolution (ranging from 764 × 504 to 2220 × 1230 pixels) in JPG or BMP format for subsequent analysis.

Routine clinical data were extracted from electronic medical records. Based on prior literature, clinical expertise, and practical considerations, 50 routine variables were initially selected (see **Supplementary Table S1**). Variables with more than 20% missing data, including D-dimer, FOBT, CRP, GFR, and HbA1c, were excluded (see **Supplementary Figure S1**). Consequently, 45 clinical variables were retained for model development.

**Figure 1:**
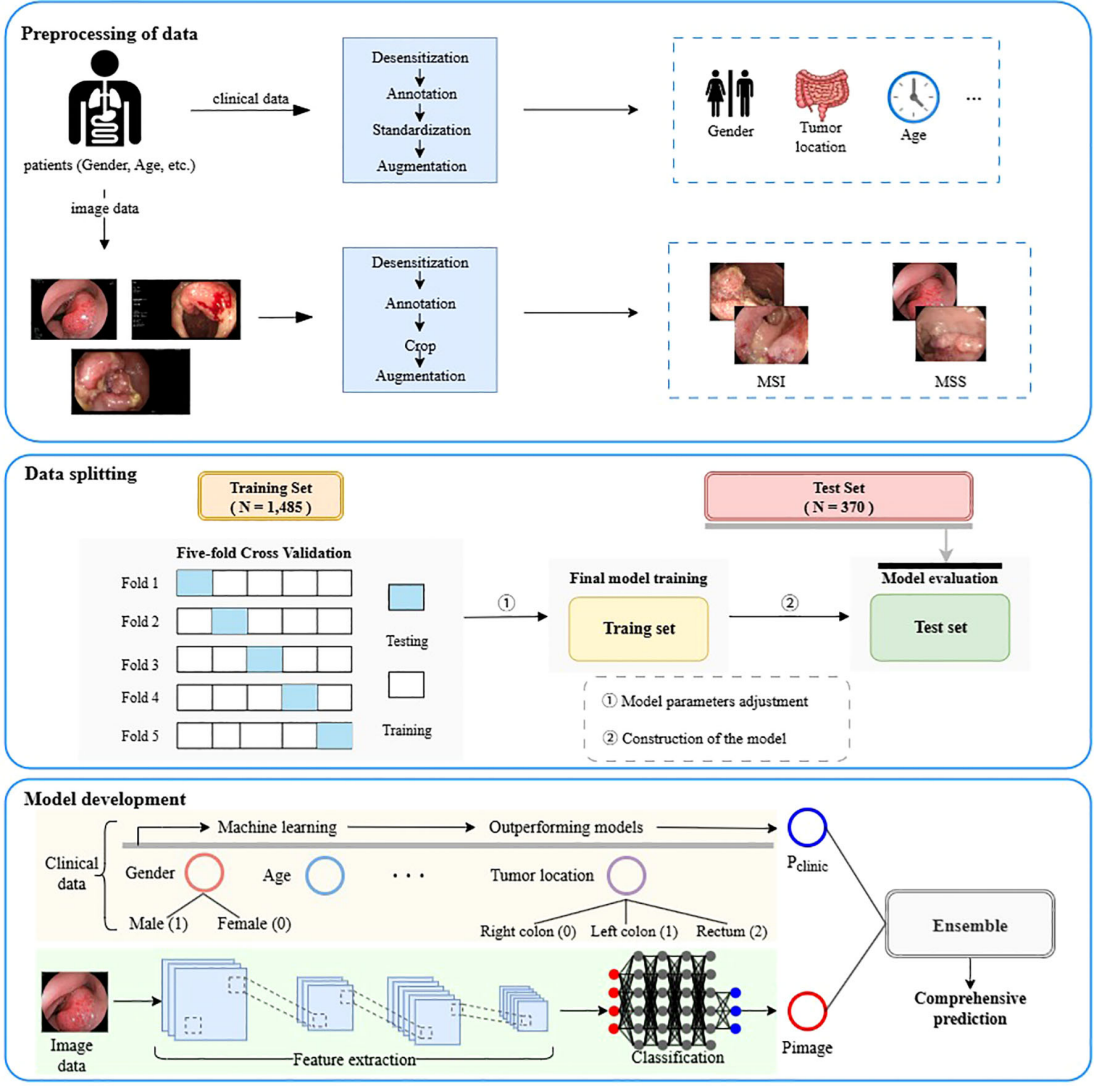
Overview of the study workflow, including data preprocessing, model development, and ensemble integration.

**Figure 2:**
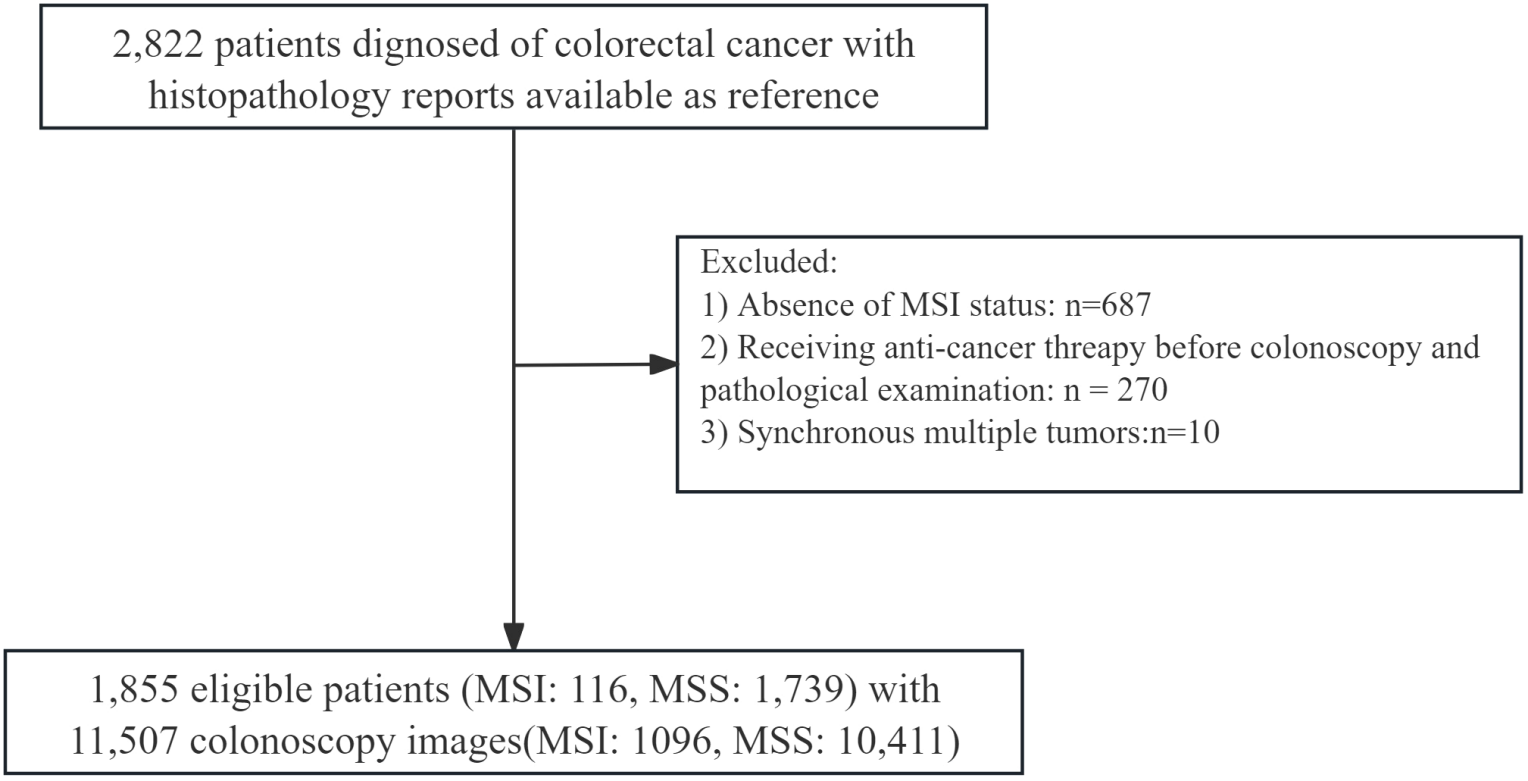
Flowchart of patient inclusion and exclusion.

In total, 1,855 patients met the inclusion criteria, including 116 MSI and 1,739 MSS cases. Pretreatment colonoscopy images were available for 1,224 patients (10,411 MSS images and 1,096 MSI images), who were included in the image-based analyses. The workflow is shown in **Figure 1** abd the overall process of patient screening and cohort inclusion is summarized in **Figure 2**.

The study protocol was approved by the Institutional Review Board (IRB) of the Nanjing Hospital of Chinese Medicine (Approval No. KY2024090), and the requirement for written informed consent was waived due to the retrospective nature of the study. All patient data were anonymized prior to analysis.

### 2.2 Data preprocessing

#### 2.2.1 Colonoscopy images

Colonoscopy images went through an initial quality control step to exclude frames that were blurred, overexposed, narrow-band imaging (NBI)—based, or showed inadequate bowel preparation. To eliminate non-informative content, we automatically removed black borders and on-screen text using a pixel-intensity–based cropping algorithm. Each image was first converted to grayscale, and every row and column was scanned to identify the first and last positions where at least 20% of the pixels fell within a valid intensity range (5–250). This threshold reliably distinguished the circular endoscopic field from the surrounding dark margins. The bounding box defined by these boundaries was then applied to the original RGB image to produce a clean, content-focused crop. The resulting images were then resized to 224 × 224 pixels to match the deep learning model’s input requirements.

#### 2.2.2 Clinical data

Outliers in continuous variables were identified using fences in the interquartile range (IQR) (Q1−1.5×IQR, Q3+1.5×IQR) and compared to the nearest bound prior to imputation. Missing values were imputed using the Multivariate Imputation by Chained Equations (MICE) method. After imputation, categorical variables were transformed using one-hot encoding, and all variables were then standardized using z-score normalization.

#### 2.2.3 Data splitting and augmentation

The dataset was split at the patient level into training (80%) and test (20%) sets with stratified sampling to preserve MSI/MSS ratios. The prevalence of MSI in our dataset was 6.29%, reflecting its relatively low frequency in colorectal cancer. Such class imbalance poses a significant challenge for modelling, as it may bias predictions toward the majority class (MSS) while underrepresenting the minority class (MSI). This imbalance can result in reduced sensitivity for MSI detection, impaired generalization, and misleading performance metrics. Addressing this issue is therefore critical to ensure that models achieve balanced performance across both classes.

To mitigate the impact of class imbalance and improve model robustness, data augmentation strategies were applied selectively to the minority class. For colonoscopy images, augmentation was performed exclusively on MSI samples during training to avoid further widening the gap between class sizes. The augmentation pipeline included random resized cropping, horizontal and vertical flipping, small rotations (± 15°>), color jittering, perspective distortion, Gaussian blur, and random erasing, thereby enhancing resilience to variability introduced by different imaging devices and acquisition conditions. For the clinical data, the Synthetic Minority Over-sampling Technique (SMOTE) was applied to the training set to generate synthetic MSI cases by interpolating between existing minority-class samples. This approach increased representation of MSI without duplicating data and preserved the underlying feature distribution, thus improving the model’s ability to recognize minority-class patterns.

### 2.3 Model development

#### 2.3.1 Image-based model

For image-based MSI status prediction, we implemented a flexible deep learning framework that supports a range of backbone architectures, including ResNet, EfficientNet, ViT, DenseNet, and VGG. All models were initialized with ImageNet-pretrained weights, with the final classification layer modified to match the binary output space. Images were resized to 224×224 pixels and normalized by the ImageNet-specific preprocessing (26). Optimization was conducted using Adam, AdamW, or stochastic gradient descent (SGD) with momentum, with the default learning rate set to $1\times 10^{-3}$.

To address the substantial class imbalance between MSI and MSS images, we initially evaluated several imbalance-aware loss functions, including focal loss (27), Tversky loss (28), focal Tversky loss (29), soft *F_β_* loss (30), and soft precision–recall penalties (31). These losses enhance minority-class learning by down-weighting easy majority-class samples, penalizing false negatives more strongly, or directly optimizing recall-oriented objectives. Although these approaches resulted in moderate performance gains, particularly in recall, the most substantial improvements were observed after applying targeted data augmentation to increase the diversity of MSI samples. After augmentation, standard binary cross-entropy provided the most stable and robust performance; therefore, all final models in this study were trained using binary cross-entropy.

#### 2.3.2 Clinical data–based machine learning model

For the tabular clinical features, we implemented a modular machine learning classification framework that supports four widely used algorithms: logistic regression (LR), support vector machine (SVM), random forest (RF), and gradient boosting classifier (GBC). All models were designed to output probabilistic predictions, thereby enabling downstream ensemble learning and calibration for clinical interpretability.

To address the class imbalance between MSI and MSS samples, we initially experimented with a range of class-weight configurations across all algorithms (for example, assigning higher penalties to the minority MSI class). However, empirical evaluation showed that class weighting did not improve model performance and, in some cases, slightly reduced it. We therefore adopted a data-level strategy and applied SMOTE to generate synthetic MSI samples during training. After SMOTE, unweighted versions of all classifiers yielded the most stable and robust performance; accordingly, all final clinical models in this study were trained using SMOTE-augmented data with unweighted class settings.

#### 2.3.3 Training detail

Model training was conducted in two stages. First, stratified five-fold cross-validation was employed to identify the optimal key hyperparameters, including learning rate, number of training epochs, optimizer choice, and convergence settings, enhancing the balance between predictive performance and generalizability. All image-based and clinical models were trained independently during this stage, without any parameter sharing or joint optimization.

For image-based models, the final configuration adopted for training employed the stochastic gradient descent (SGD) optimizer (learning rate $=1\times 10^{-3},$ batch size = 128), binary cross-entropy loss, and early stopping with a patience of three epochs based on validation loss. All image models were implemented in PyTorch (v2.7.1) and trained on an NVIDIA GeForce RTX 4070 Ti Super GPU.

For clinical tabular features, LR and SVM models were optimized with a stringent convergence tolerance ( $1\times 10^{-5}$), and the maximum number of iterations for LR was set to $1\times 10^{6}$ to ensure convergence stability. RF and GBC classifiers were trained with 200 estimators to achieve a balance between computational efficiency and predictive accuracy.

The optimal hyperparameters identified through cross-validation were used to retrain each model on the full training set. All models were trained independently, with no joint optimization or shared fine-tuning, and the resulting classifiers were then integrated using an ensemble learning strategy to generate the final multimodal predictions.

### 2.4 Multimodal ensemble integration

For ensemble learning, model selection was guided by multiple performance metrics, including accuracy, AUC, precision, and recall (32). For image–based prediction, VGG16, ViT, EfficientNet, and ResNet50 were chosen, as each demonstrated strengths across different evaluation criteria (see Section 3.2 for detailed results). For clinical tabular features, LR, GBC, and RF emerged as the top-performing models (see Section 3.3 for detailed results).

The ensemble was constructed in a *post-hoc* manner: once all selected models were fully trained, their predicted probabilities were aggregated using a probability-based majority voting strategy. Unlike hard voting, this approach integrates the calibrated probability outputs of individual models, allowing more nuanced decision-making and reducing the risk of dominance by any single classifier. In addition to soft probability-based majority voting, we also implemented a stacking ensemble using a multi-layer perceptron (MLP) meta-classifier. Stacking results were evaluated but not adopted, as it consistently demonstrated substantially lower recall for the minority MSI class. Full stacking performance is reported in **Supplementary Table S2**.

## 3 Results

### 3.1 Study cohort characteristics

Among the 1,844 patients (MSI = 113; MSS = 1,731), MSI cases were significantly younger (median 60 vs. 66 years; *p* = 0.004) and more frequently located in the right colon (51.3% vs. 14.8%; *p <* 0.001). MSI patients had a lower prevalence of hypertension (32.7% vs. 44.6%; *p* = 0.014) and exhibited lower CEA (*p <* 0.001). Hematologic indices revealed lower HGB, MCV, pLYM, and cLYM, with higher RDW and pNEUT in MSI cases (all *p <* 0.001). In addition, MSI tumours were associated with lower bilirubin and lipid levels (e.g., LDL; *p <* 0.05). These findings are consistent with the distinct clinical and biological profile of MSI colorectal cancer (33). Baseline characteristics of the cohort are summarized in **Table 1**.

**Table 1:** Baseline characteristics of the study cohort.

Characteristic	Overall (N = 1,844)	MSI (N = 113)	MSS (N = 1,731)	P-value
Gender				0.098
Female	729 (39.5%)	53 (46.9%)	676 (39.1%)	
Male	1,115 (60.5%)	60 (53.1%)	1,055 (60.9%)	
Age	66 (58, 73)	60 (50, 72)	66 (58, 73)	0.004
Other primary tumor	76 (4.1%)	9 (8.0%)	67 (3.9%)	0.047
Hypertension	799 (43.8%)	37 (32.7%)	762 (44.6%)	0.014
Diabetes	303 (16.6%)	12 (10.6%)	291 (17.0%)	0.076
Family history of tumor	29 (1.6%)	3 (2.7%)	26 (1.5%)	0.419
Smoking	497 (27.0%)	27 (23.9%)	470 (27.2%)	0.441
Drinking	378 (20.5%)	16 (14.2%)	362 (21.0%)	0.083
Height	165 (160,170)	165 (160,170)	165 (160,170)	0.874
Weight	65 (57,72)	62 (55,70)	65 (57.5,72)	0.056
BMI	23.6 (21.5,25.7)	23.1 (20.9,25.2)	23.6 (21.6,25.8)	0.033
				*<* 0.001
Left colon	560 (30.4%)	30 (26.6%)	530 (30.6%)	
Rectum	969 (52.6%)	25 (22.1%)	944 (54.6%)	
Right colon	314 (17.0%)	58 (51.3%)	256 (14.8%)	
AFP	2.58 (1.90,3.57)	2.36 (1.74,3.15)	2.60 (1.92,3.59)	0.036
CEA	3.80 (2.16,8.60)	2.50 (1.51,5.15)	3.86 (2.24,8.78)	*<* 0.001
CA125	9.75 (6.92,13.97)	10.63 (8.03,14.95)	9.60 (6.88,13.96)	0.018
CA199	12.06 (7.29,21.68)	10.96 (6.58,17.37)	12.15 (7.30,21.88)	0.174
CRP	4.00 (2.00,12.00)	9.35 (3.00,27.80)	3.50 (2.00,10.00)	*<* 0.001
WBC	6.10 (5.00,7.40)	5.90 (5.00,7.70)	6.10 (5.02,7.32)	0.626
pNEUT	61.8 (55.9,68.5)	64.4 (58.8,71.6)	61.4 (55.6,68.3)	*<* 0.001
pLYM	27.4 (21.3,33.0)	24.5 (19.8,30.3)	27.5 (21.5,33.3)	0.001
pMONO	6.40 (5.40,7.50)	6.50 (5.60,7.40)	6.40 (5.40,7.50)	0.429
cNEUT	3.70 (2.90,4.77)	3.79 (3.01,5.04)	3.70 (2.90,4.74)	0.376
cLYM	1.60 (1.26,2.00)	1.43 (1.13,1.83)	1.61 (1.27,2.02)	0.003
cMONO	0.39 (0.31,0.49)	0.39 (0.30,0.53)	0.39 (0.31,0.49)	0.504
RBC	4.22 (3.87,4.57)	4.10 (3.68,4.34)	4.23 (3.88,4.59)	*<* 0.001
HGB	126 (111,137)	114 (91,127)	126 (112,138)	*<* 0.001
MCV	90.1 (85.7,93.5)	87.4 (78.8,91.1)	90.3 (85.9,93.6)	*<* 0.001
RDW	13.3 (12.8,14.1)	13.6 (13.0,15.3)	13.3 (12.8,14.1)	*<* 0.001
PLT	212 (166,266)	221 (152,281)	211 (166,264)	0.645
ALT	15 (10,21)	12 (9,18)	15 (10,21)	0.001
AST	16 (13,21)	15 (11.5,19)	16 (13,21)	0.004
TP	64.9 (61.5,69.0)	64.9 (60.9,69.1)	64.9 (61.6,68.9)	0.727
ALB	38.2 (35.7,40.8)	37.5 (34.8,39.8)	38.2 (35.7,40.9)	0.027
GLB	27.1 (24.3,30.1)	27.8 (24.4,31.1)	27.0 (24.3,30.0)	0.096
A/G ratio	1.38 (1.10,1.55)	1.32 (1.00,1.49)	1.39 (1.11,1.56)	0.020
TBil	11.3 (8.4,14.9)	9.7 (6.7,12.5)	11.3 (8.5,15.0)	*<* 0.001
DBil	3.00 (2.20,4.00)	2.50 (2.00,3.50)	3.00 (2.20,4.00)	0.001
IBil	8.10 (5.90,11.00)	7.20 (4.90,9.10)	8.20 (6.05,11.15)	*<* 0.001
Glu	5.19 (4.68,5.97)	5.09 (4.75,5.92)	5.19 (4.67,5.97)	0.722
TC	4.59 (3.98,5.23)	4.29 (3.80,5.04)	4.60 (4.00,5.25)	0.006
TG	1.18 (0.88,1.62)	1.09 (0.82,1.50)	1.19 (0.89,1.63)	0.064
HDL	1.16 (1.00,1.36)	1.08 (0.93,1.33)	1.16 (1.00,1.36)	0.011
LDL	2.63 (2.17,3.13)	2.41 (2.05,2.93)	2.64 (2.19,3.14)	0.027
Urea	5.50 (4.55,6.70)	5.22 (3.99,6.19)	5.52 (4.58,6.73)	0.005
Crea	66.0 (56.0,78.0)	64.0 (50.9,75.0)	67.0 (56.0,78.0)	0.054
UA	302 (245,368)	292 (225,363)	303 (246,368)	0.159
GFR	98 (89,104)	99 (90,107)	98 (89,104)	0.396
FOBT				0.801
Negative	89 (8.2%)	5 (7.5%)	84 (8.3%)	
Positive	985 (91.8%)	62 (92.5%)	923 (91.7%)	

*Statistical tests:* Pearson’s Chi-squared test, Wilcoxon rank-sum test, Fisher’s exact test.

Continuous variables are expressed as median (IQR). MSI, microsatellite instability; MSS, microsatellite stable.

### 3.2 Image-based deep learning model evaluation

We trained and evaluated five deep learning architectures on colonoscopy images for MSI prediction: ResNet50, EfficientNet, DenseNet, VGG16, and ViT. As summarized in **Table 2**, all models demonstrated good discriminative ability, with AUROC values ranging from 0.873 to 0.896. VGG16 achieved the best overall balance, with the highest accuracy (0.832), precision of 0.943, recall of 0.708, and an AUROC of 0.894. ViT showed comparable performance, achieving the highest recall (0.721) and the best AUROC (0.896), although with slightly lower precision (0.911). ResNet50 and EfficientNet reached very high precision (0.955 and 0.963, respectively) but lower recall (∼ 0.68), indicating that their positive predictions were highly reliable but more conservative, potentially missing MSI cases. DenseNet also performed well (accuracy 0.818, AUROC 0.891, precision 0.941, recall 0.678), though it did not outperform VGG16 or ViT.

**Table 2:** Performance of image-based deep learning classifiers for MSI prediction.

Classifier	Accuracy	Precision	Recall	AUROC
ResNet-50	0.825	0.955	0.681	0.873
EfficientNet	0.825	**0.963**	0.675	0.877
DenseNet	0.818	0.941	0.678	0.891
VGG-16	**0.832**	0.943	0.708	0.894
Vision Transformer (ViT)	0.825	0.911	**0.721**	**0.896**

Results are reported on the independent test set. Best results for each metric are highlighted in bold.

**Figure 3:**
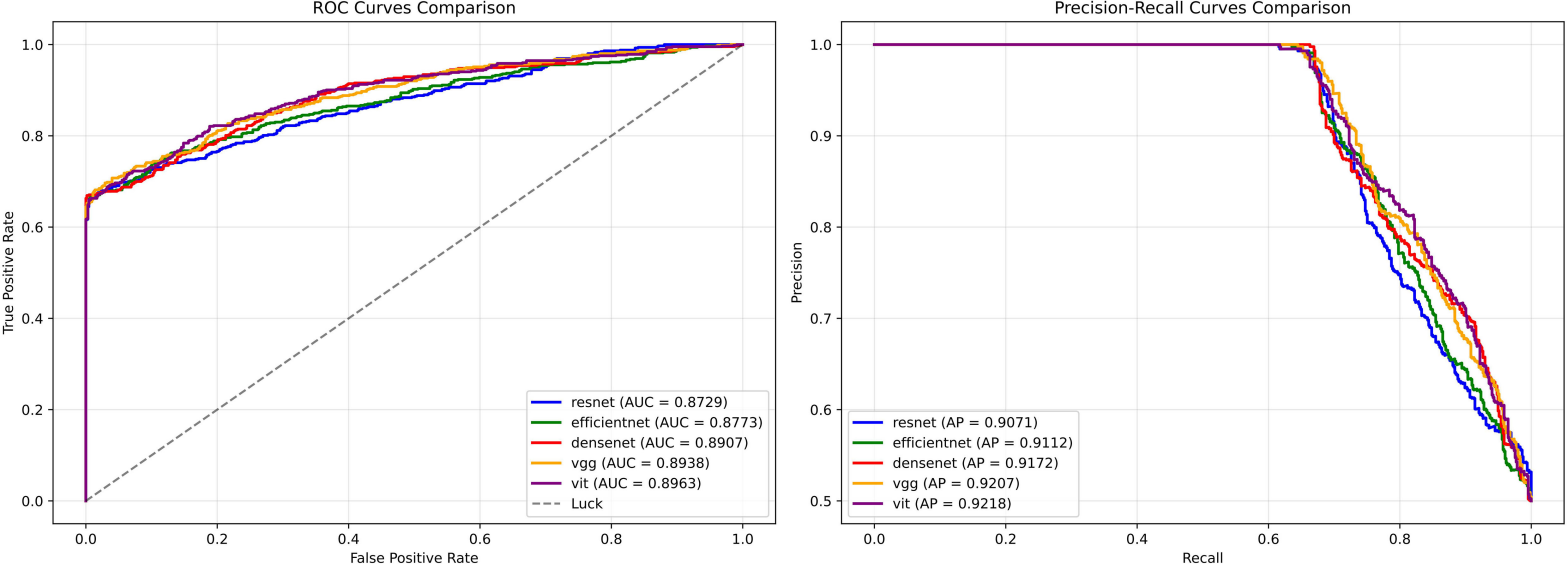
Receiver operating characteristic (ROC) and precision–recall (PR) curves for the five image-based deep learning models. The ROC (left) and PR (right) curves illustrate the discriminative performance of five convolutional architectures (ResNet-50, EfficientNet, DenseNet, VGG-16, and Vision Transformer).

Receiver operating characteristic (ROC) and precision–recall (PR) curves for these models are provided in **Figure 3**, which further illustrate these trade-offs. Specifically, ResNet50 and EfficientNet emphasize conservative, high-precision predictions, while VGG16 and ViT demonstrate a more favorable balance between sensitivity and specificity. Collectively, these findings confirm that image-based deep learning models can effectively discriminate MSI from MSS, with VGG16 and ViT offering the most clinically relevant performance, and ResNet50 and EfficientNet contributing complementary high-precision predictors.

### 3.3 Clinical data–based machine learning model evaluation

We compared the performance of four machine learning classifiers trained on routine clinical variables: LR, SVM, RF, and GBC. As summarized in **Table 3**, LR achieved the most balanced performance with an accuracy of 0.825 and an AUROC of 0.898. Tree-based models (RF and GBC) and SVM demonstrated higher precision (≥ 0.93) but substantially lower recall (0.55–0.60), indicating that they identified fewer true MSI cases despite fewer false positives. In contrast, LR maintained a favorable trade-off between precision (0.823) and recall (0.828), suggesting superior sensitivity for MSI detection.

**Table 3:** Performance of clinical data–based machine learning classifiers for MSI prediction.

Classifier	Accuracy	Precision	Recall	AUROC
Logistic Regression (LR)	**0.825**	0.823	**0.828**	0.898
Support Vector Machine (SVM)	0.768	0.932	0.579	**0.943**
Random Forest (RF)	0.757	0.938	0.551	0.940
Gradient Boosting Classifier (GBC)	0.781	**0.946**	0.596	0.939

Model performance was evaluated on the independent test set. Best results for each metric are in bold.

**Figure 4:**
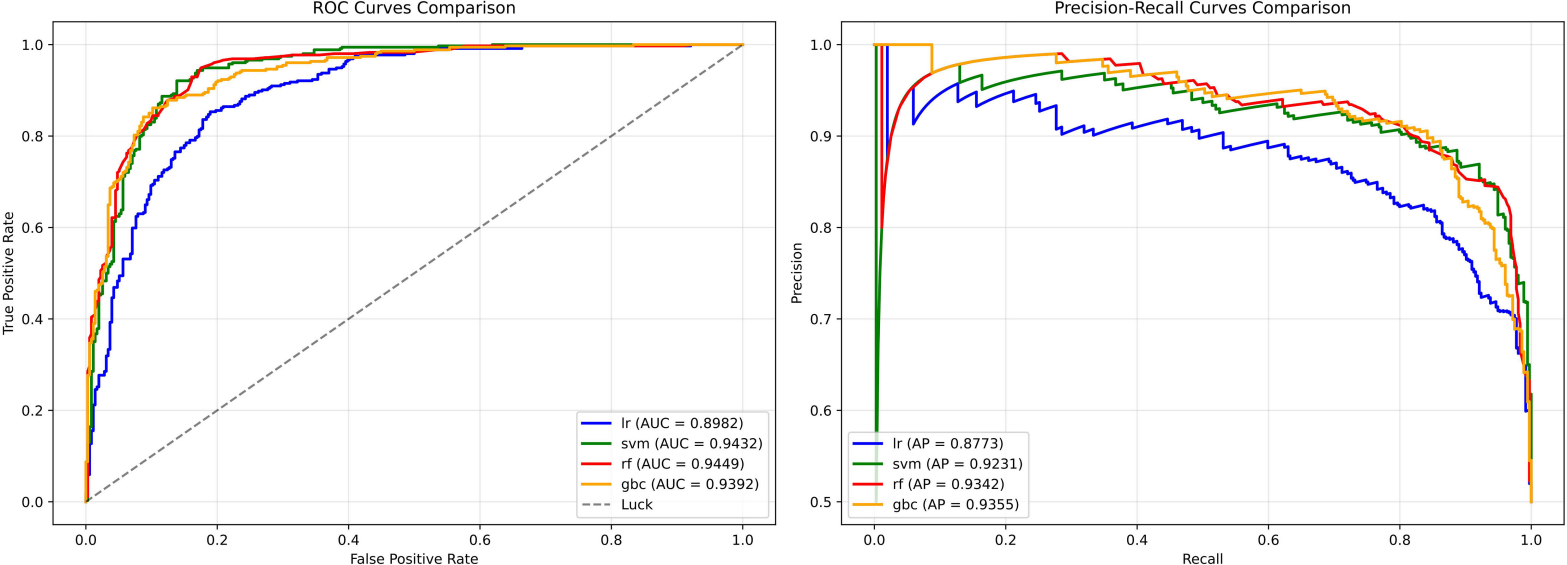
Receiver operating characteristic (ROC) and precision–recall (PR) curves for the four clinical data–based machine learning models. The ROC (left) and PR (right) curves compare four classifiers trained on clinical variables: Logistic Regression (LR), Support Vector Machine (SVM), Random Forest (RF), and Gradient Boosting Classifier (GBC).

ROC and PR curves for these classifiers are provided in **Figure 4**. These visualizations further illustrate the trade-offs between model sensitivity and specificity, showing that while RF, GBC, and SVM achieved strong discriminative capability, LR provided the most robust and clinically practical performance by balancing precision and recall across the decision threshold.

### 3.4 Comparative performance across modalities

To better understand the complementary contributions of colonoscopy image–based deep learning models and clinical data–based machine learning models, we conducted a comparative analysis across the two modalities.

The image-based models demonstrated relatively higher recall for MSI prediction. For example, VGG-16 and ViT achieved recalls of 0.708 and 0.721, respectively, reflecting the ability of deep networks to capture fine-grained morphological patterns such as mucosal irregularity, glandular disruption, and abnormal vascularity. These cues appear particularly informative for detecting MSI tumors. However, the AUROC values of image models (0.873–0.896) were modestly lower than those of the best clinical models, indicating greater variability in overall discrimination.

In contrast, the clinical machine learning models achieved higher AUROC but lower recall. Logistic Regression obtained the highest AUROC (0.898) and showed stable precision, highlighting the structured and biologically informative nature of clinical variables such as biochemical markers, hematologic indices, and patient demographics. However, these models were more conservative in predicting the MSI class, resulting in lower sensitivity. This pattern suggests that clinical variables better support global discrimination but are less effective at identifying minority-class MSI cases.

Taken together, these results demonstrate that each modality captures distinct and complementary aspects of MSI biology. Image-based models excel in sensitivity by detecting subtle morphological alterations, whereas clinical models provide stronger overall discrimination but miss more MSI cases. By integrating high-recall image predictors with high-AUROC clinical predictors, the ensemble achieves improved robustness and sensitivity compared with either modality alone.

### 3.5 Ensemble model evaluation

Using a majority voting strategy, we evaluated multiple combinations of clinical (LR, GBC, RF) and image-based (VGG16, ViT, ResNet50, EfficientNet, DenseNet) models (**Table 4**). Overall, ensembles consistently outperformed most single models in terms of balanced accuracy and recall. The best-performing ensembles included both clinical and image models, particularly LR + RF + ResNet + ViT + VGG + EfficientNet, which achieved the highest accuracy (0.886), recall (0.845), and AUROC (0.886) while maintaining high precision (0.920). Similar performance was observed when DenseNet was additionally included, suggesting that adding further redundant models did not provide incremental benefit. In contrast, smaller ensembles (e.g., LR + GBC + ViT + VGG) achieved lower recall (0.734), underscoring the importance of including diverse architectures.

**Table 4:** Performance of ensemble models integrating clinical and image-based predictors for MSI prediction.

Ensemble model	Accuracy	Precision	Recall	AUROC
Ensemble-1	0.840	0.932	0.734	0.840
Ensemble-2	0.883	0.920	0.839	0.883
Ensemble-3	0.883	0.920	0.839	0.883
Ensemble-4	0.843	0.932	0.740	0.843
Ensemble-5	0.840	**0.948**	0.720	0.840
Ensemble-6	**0.886**	0.920	**0.845**	**0.886**
Ensemble-7	0.877	0.913	0.833	0.877

All results are reported on the independent test set. Best results for each metric are highlighted in bold.

Ensemble configurations combine clinical and image-based models as follows: Ensemble-1 = LR + GBC + ViT + VGG; Ensemble-2 = LR + RF + ViT + VGG; Ensemble-3 = LR + RF + ResNet + ViT + VGG; Ensemble-4 = LR + GBC + ResNet + ViT + VGG; Ensemble-5 = LR + GBC + RF + ResNet + ViT + VGG; Ensemble-6 = LR + RF + ResNet + ViT + VGG + EfficientNet Ensemble–7 = LR + RF + ResNet + ViT + VGG + EfficientNet + DenseNet.

Collectively, these findings indicate that integrating both clinical and image-based models provides more robust and reliable predictions of MSI status than individual models, with majority voting effectively balancing sensitivity and specificity. For completeness, we also evaluated stacking ensembles using the same model combinations. However, stacking yielded lower performance, especially in recall (0.54–0.60), and therefore was not selected as the primary integration strategy. Detailed stacking results are provided in **Supplementary Table S2**.

### 3.6 Interpretability analysis

To enhance transparency and clinical relevance, interpretability analyses were conducted for both image and clinical models. For deep learning models, Grad-CAM was applied to visualize salient image regions most influential in MSI predictions, allowing qualitative assessment of whether the model attended to relevant mucosal and vascular patterns. For clinical machine learning models, SHAP were used to quantify the contribution of each variable to predictions. SHAP values provided both global feature importance rankings and local instance-level explanations.

**Figure 5:**
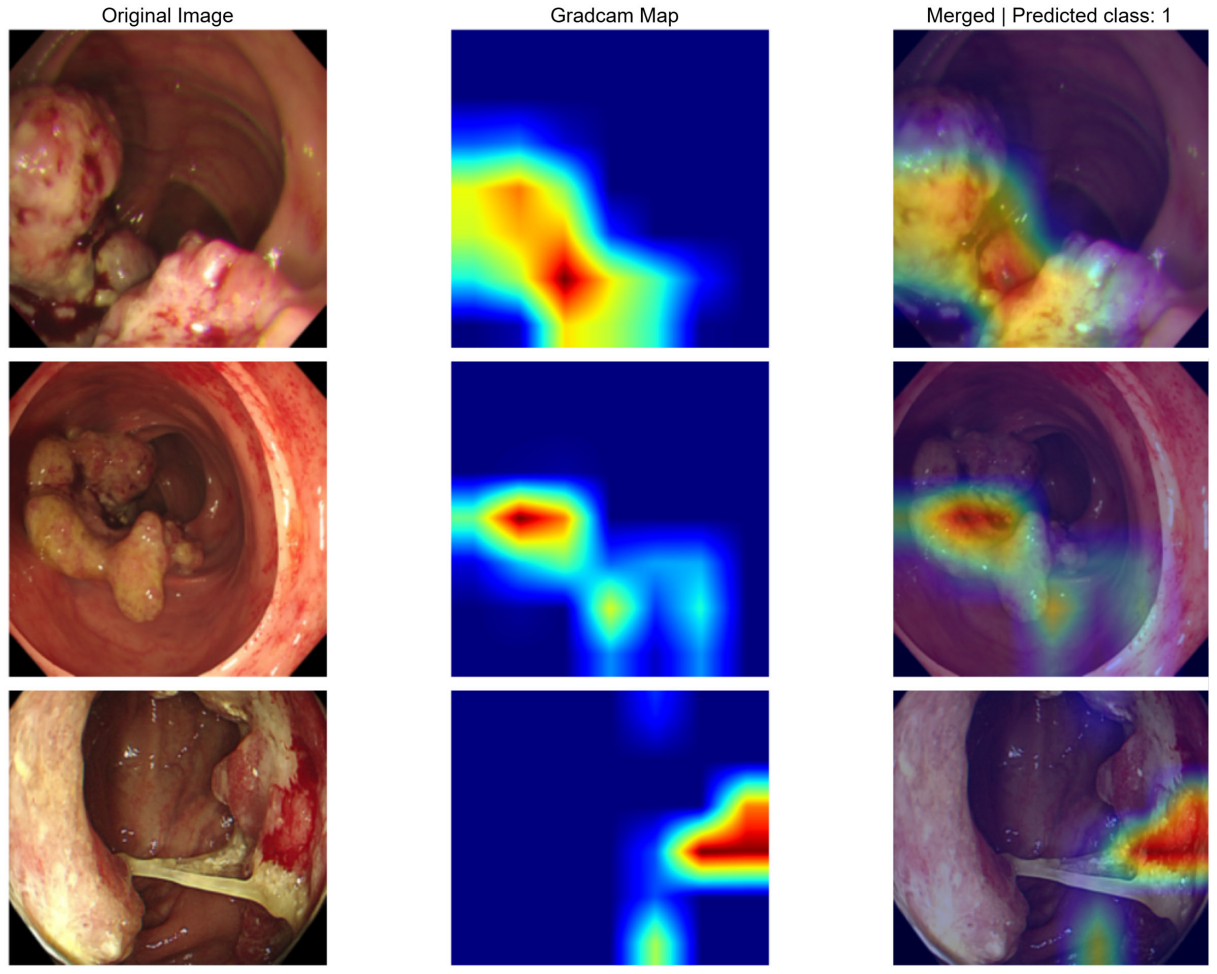
Representative Grad-CAM visualizations for MSS colonoscopy images. Each row shows the original image (left), the Grad-CAM activation map (middle), and the merged overlay (right).

**Figure 6:**
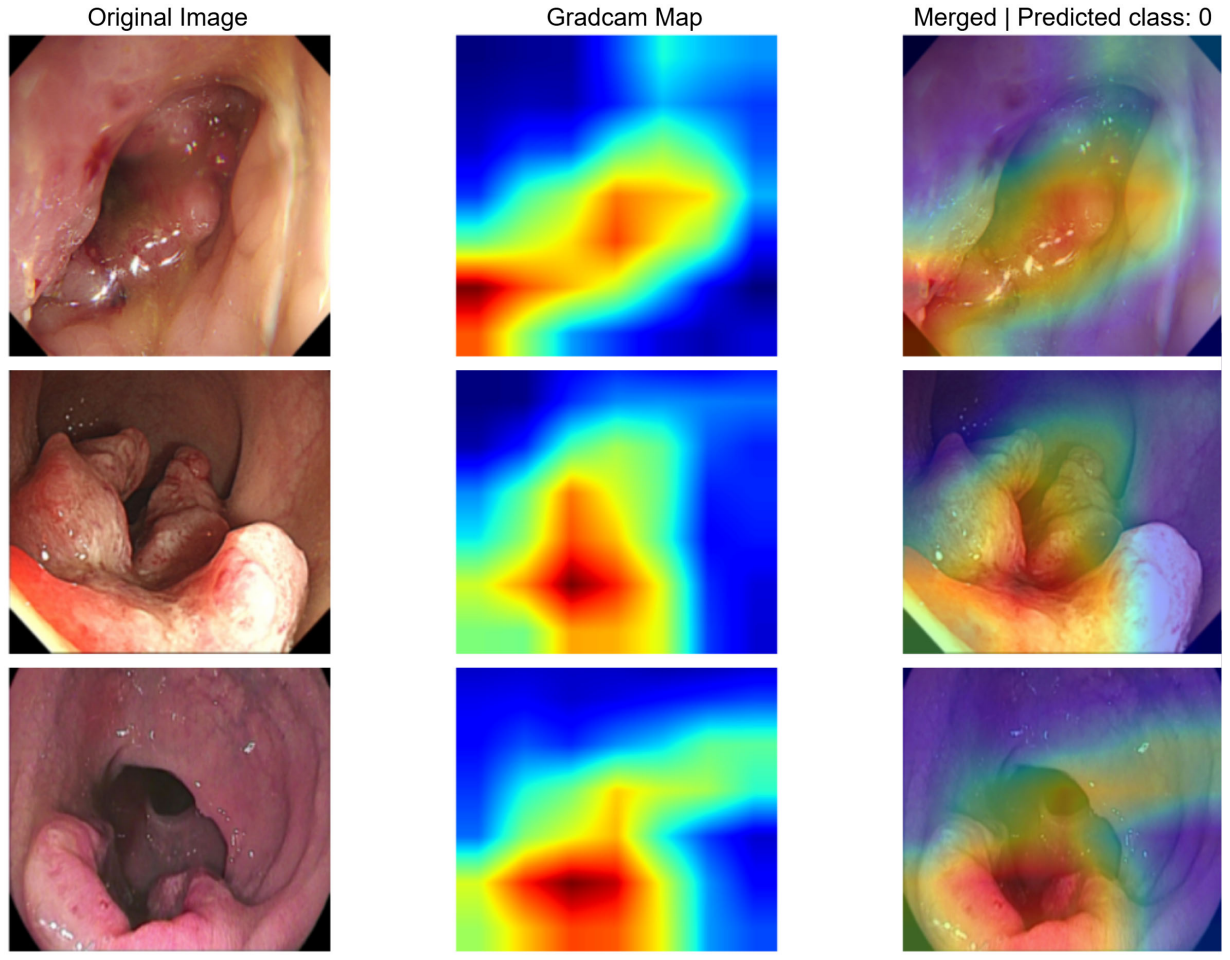
Representative Grad-CAM visualizations for MSI colonoscopy images. Each row shows the original image (left), the Grad-CAM activation map (middle), and the merged overlay (right).

For the image models, Grad-CAM visualization revealed that the networks primarily focused on tumor regions and surrounding mucosal structures when generating predictions. In MSS cases, highlighted areas tended to align with the tumor bulk and adjacent mucosa (**Figure 5**), whereas in MSI cases, attention maps often emphasized irregular lesion borders and heterogeneous mucosal patterns (**Figure 6**). These findings suggest that the models leveraged clinically plausible visual cues consistent with endoscopic examination, thereby supporting the biological interpretability of the predictions.

**Figure 7:**
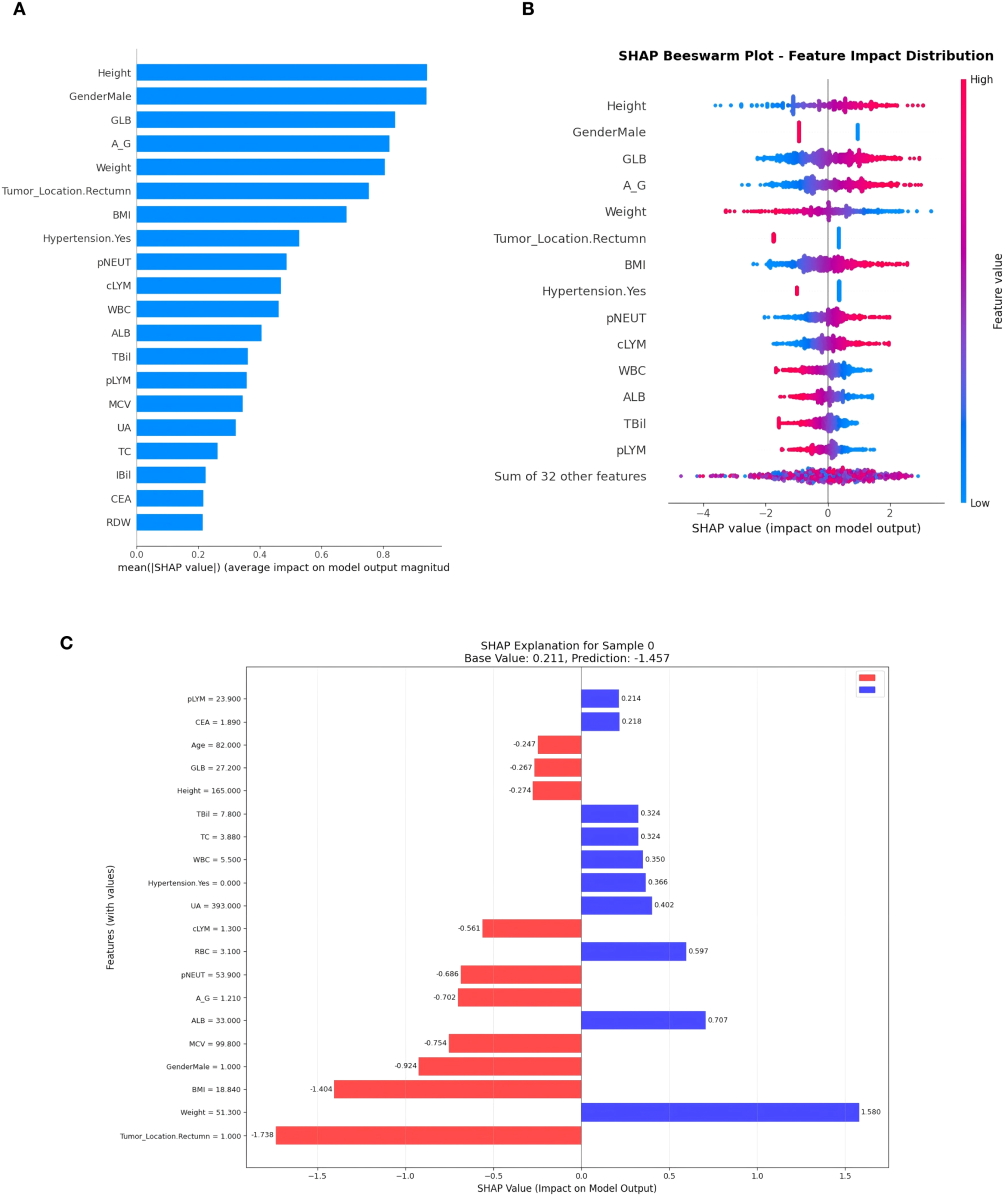
SHAP-based interpretability analysis of the clinical model for MSI prediction. **(A)** Global feature importance ranked by mean absolute SHAP value. **(B)** SHAP beeswarm plot showing the distribution and direction of feature effects across all patients; red represents higher feature values and blue lower values. **(C)** Example of an individual patient’s SHAP explanation (waterfall plot), illustrating how specific feature values increased (blue) or decreased (red) the predicted probability of MSI.

For the clinical models, SHAP analysis identified both demographic and clinical factors as key predictors of MSI. Height, gender, GLB, A/G ratio, weight, and tumor location emerged as the most influential features, with additional contributions from BMI, hypertension, and peripheral blood indices such as pNEUT and cLYM (**Figure 7a**). The beeswarm plots highlighted patient-level heterogeneity, with certain variables (e.g., tumor location and anthropometric measures) exerting consistent directional effects, whereas others showed more variable impacts (**Figure 7b**). At the individual patient level, waterfall plots demonstrated how combinations of features synergistically increased or decreased the likelihood of MSI prediction, providing a transparent rationale for model outputs (**Figure 7c**).

Together, the interpretability analyses confirm that both clinical and image-based models captured meaningful features, enhancing trust in the ensemble framework by linking predictive signals to clinically relevant patterns.

## 4 Discussion

In this study, we developed and evaluated a multimodal ensemble model integrating colonoscopy images and clinical data for MSI prediction in colorectal cancer. Both image and clinical-based models showed strong discriminative performance, with VGG16 excelling among image models, and LR performing best among clinical models. Importantly, a majority-voting ensemble combining image and clinical data achieved better performance than single models.

Prior work has shown that MSI can be predicted from pathology and radiology images, including H&E whole-slide models such as WiseMSI (34) and CT/MRI radiomics approaches (35, 36). While these imaging modalities have demonstrated strong performance, they typically require tissue sampling, specialized scanners, or labor-intensive tumor segmentation, which may limit scalability in routine clinical practice. In contrast, colonoscopy is widely available, non-invasive, and performed before treatment in nearly all patients, making it a practical platform for real-time MSI risk stratification.

Colonoscopy has been explored as a promising modality for MSI prediction in colorectal cancer. Lo et al. (19) applied a Vision Transformer to data from 441 patients (34 MSI, 407 MSS), achieving an AUC of 0.86, with a sensitivity of 0.47 and a specificity of 0.94. Cai et al. (18) subsequently developed MMR-Scopy, a ResNet50-based model trained on 5,226 colonoscopy images, which achieved an AUROC of 0.948 in the internal test set and 0.807 in external validation, with a sensitivity of 0.796 and a specificity of 0.670. In contrast to these unimodal approaches, our study leveraged a substantially larger cohort of 1,844 patients (113 MSI, 1,731 MSS) and 11,507 colonoscopy images, providing greater statistical power and model robustness. Furthermore, we extended the framework beyond image-only prediction to multimodal integration. By combining image-based deep learning with clinical data–based machine learning, our ensemble model outperformed individual modalities, achieving higher precision (0.920) and recall (0.845) and thereby improving the identification of MSI cases.

Although some single models (such as ViT and LR) achieved slightly higher AUROC scores than the ensemble, this difference is partly attributable to class imbalance. AUROC is relatively insensitive to minority-class performance (37), and conservative models such as LR or models that capture strong morphological cues such as ViT may appear to perform better under AUROC even though they miss a greater number of MSI cases. In contrast, the ensemble consistently achieved substantially higher recall, reflecting its ability to integrate complementary strengths from both image-based and clinical models. This trade-off is expected because the ensemble focuses on balanced performance rather than maximizing a single discrimination metric. Recall is especially important in MSI screening, because missed MSI cases can delay immunotherapy eligibility or reduce the likelihood of identifying patients with Lynch syndrome. Therefore, the ensemble’s improved sensitivity is more aligned with real-world clinical priorities.

We also evaluated more complex integration strategies, including stacking with an MLP meta-classifier. However, stacking consistently resulted in substantially lower recall. This likely occurs because the meta-model inherits the class imbalance present in the training data, which biases predictions toward the majority MSS class. In contrast, probability-based majority voting introduces no additional trainable parameters and therefore avoids amplifying imbalance. Its transparent combination of model probabilities further enhances interpretability in clinical settings. For completeness, the stacking results are reported in the **Supplementary Table S2**.

The strong performance of VGG-16 among image-based models and LR among clinical models can be explained by the interplay between model architecture and data characteristics. VGG16 performs well on colonoscopy images because the visual patterns relevant to MSI, including coarse mucosal textures, vascular irregularities, and tumor-surface morphology, are effectively captured by stacked 3×3 convolutions without the need for deep residual blocks. This architectural simplicity helps reduce overfitting and supports stable training on a dataset of moderate size. In contrast, the structured clinical variables exhibit largely linear or monotonic relationships with MSI status (38), which makes LR particularly appropriate. Tree-based models and SVM tended to overfit the minority MSI class or required more extensive hyperparameter tuning. For these reasons, the observed performance reflects the compatibility between each model and the underlying data rather than differences in model complexity.

A critical barrier to clinical adoption of AI models is their interpretability. We therefore conducted model explainability analyses using Grad-CAM for the image-based models and SHAP for the clinical models. The Grad-CAM visualizations of the image-based models showed that the networks attended to clinically relevant tumor regions and mucosal abnormalities in colonoscopy images, rather than being driven by irrelevant background structures. For MSI tumors, Grad-CAM maps highlighted irregular vascular patterns and mucosal disruptions. In MSS tumors, attention was more diffusely distributed but still focused on tumor mass regions. Together, these interpretability analyses not only enhance clinician trust in model predictions but also provide insight into potential endoscopic correlates of MSI biology. Histopathologically, MSI tumors are characterized by heterogeneous glandular, mucinous, and solid components, along with increased microvascular density (39). These features likely translate endoscopically into tumors with more prominent mucosal secretions and irregular, enlarged vascular patterns, which were consistently emphasized by the Grad-CAM outputs.

Our SHAP analysis revealed key determinants underlying MSI prediction. Tumor location was the most influential factor, with rectal tumors contributing negatively, consistent with the predominance of MSI in right-sided colon cancers (40). Anthropometric features such as height, weight, and BMI were also important, aligning with evidence that obesity is more strongly linked to MSS colorectal cancer (41). Gender showed a moderate effect, supporting findings that MSI-H tumors are more frequent in men, while estrogen may exert a protective role in women (42). Immune and hematologic indices (pNEUT, cLYM) reflected the immune-rich and inflammatory microenvironment of MSI tumors (43), whereas liver function arkers (ALB, GLB, A/G ratio) suggested potential metabolic associations. Although hypertension also contributed, its biological relevance remains uncertain. Together, these findings confirm that the model captured clinically plausible and biologically meaningful predictors, reinforcing its interpretability and translational potential.

Our results have several important clinical implications. MSI is a critical biomarker in CRC, with relevance for both prognosis and therapy selection, particularly response to immune checkpoint inhibitors. Conventional MSI testing relies on IHC or PCR, which are invasive, time-consuming, and costly. By leveraging colonoscopy images and routine clinical data, our approach offers a non-invasive, rapid, and cost-effective alternative for MSI pre-screening. In practice, such a system could be deployed at the time of colonoscopy, providing immediate stratification and guiding subsequent confirmatory testing. For example, patients predicted as MSS with high confidence could bypass unnecessary molecular testing, reducing diagnostic burden and cost, while MSI-positive predictions could be prioritized for confirmatory IHC or PCR. This workflow has the potential to accelerate treatment decision-making, improve resource allocation, and reduce the workload of pathologists and laboratory personnel.

Several limitations should be acknowledged. First, This retrospective single-center design may introduce selection bias and limit the generalizability of our findings. External validation using independent multi-center datasets is essential to assess generalizability and clinical applicability. Second, although the low MSI prevalence reflects real-world epidemiology, it may reduce sensitivity for minority-class detection despite augmentation and ensemble strategies. This may be because some patients in our hospital did not undergo MSI testing due to cost or other factors. Nonetheless, this further strengthens the potential of our model to pre-screen MSI status and guide decisions regarding the necessity of IHC or PCR testing. Finally, although the ensemble improves predictive balance, it introduces additional computational overhead that may affect real-time deployment during colonoscopy. Future work will explore lightweight architectures, model distillation, or on-device optimization to improve efficiency. Notably, our soft probability–based voting module itself requires minimal computation, which helps mitigate deployment challenges.

In conclusion, we demonstrate that combining colonoscopy image–based deep learning and clinical machine learning models through ensemble learning enables accurate, interpretable, and non-invasive MSI prediction. Grad-CAM and SHAP analyses enhance transparency and clinical trust by linking predictions to biologically meaningful patterns. This ensemble framework holds promise as a practical adjunct to molecular assays, with potential to streamline diagnostics, reduce testing burden, and support personalized treatment strategies in colorectal cancer.

## Data Availability

The datasets generated during and/or analyzed during the current study are available from the corresponding author on reasonable request.
