# Supplementary material for: Ensemble learning for predicting microsatellite instability in colorectal cancer using pretreatment colonoscopy images and clinical data
Source: Front Oncol. 2026 Jan 2;15:1734076. doi: 10.3389/fonc.2025.1734076 (PMC12807959; doi:10.3389/fonc.2025.1734076)
Supplement: Supplementary file 1 [file DataSheet1.pdf]

Supplementary Material

1 SUPPLEMENTARY TABLES AND FIGURES

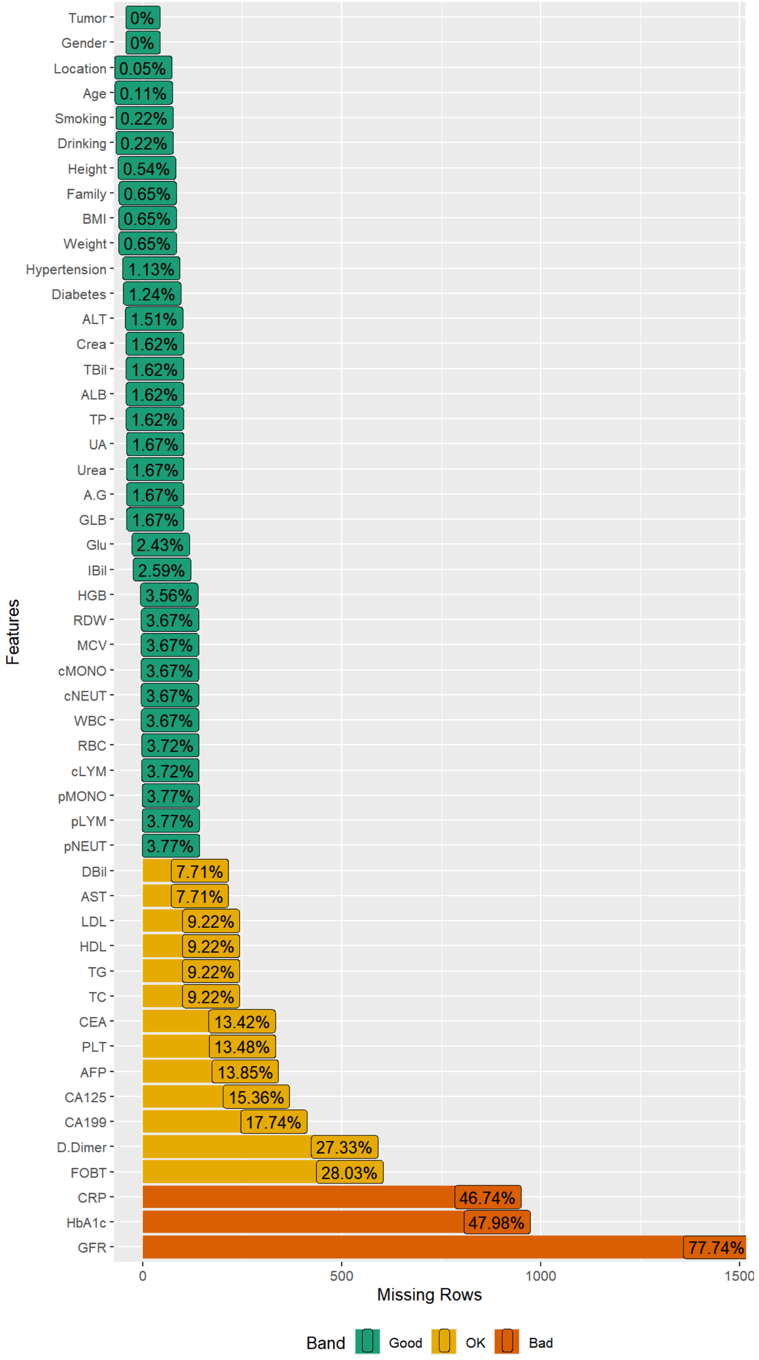

Figure S1. Proportion of missing values across all clinical variables. Each bar represents the percentage of missing data for a given feature, grouped by completeness bands: Good (<5% missing), OK (5–20% missing), and Bad (>20% missing).

Table S1. Variable definitions and data types used in the study. Continuous variables are expressed as measured values; categorical variables are encoded as indicated.

SN	Variable	Description	Type	Values / Units
1	Gender	Sex of the patient	Categorical	0 = Male; 1 = Female
2	Age	Age of the patient	Continuous	years
3	Drinking	History of alcohol consumption	Categorical	0 = No; 1 = Yes
4	Smoking	History of smoking	Categorical	0 = No; 1 = Yes
5	Diabetes	History of diabetes	Categorical	0 = No; 1 = Yes
6	Hypertension	History of hypertension	Categorical	0 = No; 1 = Yes
7	Height	Height of the patient	Continuous	cm
8	Weight	Weight of the patient	Continuous	kg
9	BMI	Body mass index	Continuous	kg/m^2
10	Family	Family history of tumor	Categorical	0 = No; 1 = Yes
11	Tumor	History of primary tumor in other systems	Categorical	0 = No; 1 = Yes
12	Tumor_Location	Location of primary cancer	Categorical	0 = Right colon; 1 = Left colon; [illegible] Rectum
13	AFP	Alpha-fetoprotein	Continuous	ng/mL
14	CEA	Carcinoembryonic antigen	Continuous	ng/mL
15	CA125	Carbohydrate antigen 125	Continuous	U/mL
16	CA199	Carbohydrate antigen 199	Continuous	U/mL
17	CRP	C-reactive protein	Continuous	mg/L
18	WBC	White blood cell	Continuous	$\times 10^9/L$
19	pNEUT	Percentage of neutrophils	Continuous	%
20	pLYM	Percentage of lymphocytes	Continuous	%
21	pMONO	Percentage of monocytes	Continuous	%
22	cNEUT	Count of neutrophils	Continuous	$\times 10^9/L$
23	cLYM	Count of lymphocytes	Continuous	$\times 10^9/L$
24	cMONO	Count of monocytes	Continuous	$\times 10^9/L$
25	RBC	Red blood cell count	Continuous	$\times 10^{12}/L$
26	HGB	Hemoglobin	Continuous	g/L
27	MCV	Mean corpuscular volume	Continuous	fL
28	RDW	Red cell distribution width	Continuous	%
29	PLT	Platelet count	Continuous	$\times 10^9/L$
30	HbA1c	Glycated hemoglobin A1c	Continuous	%
31	D-Dimer	D-dimer	Continuous	g/mL
32	ALT	Alanine aminotransferase	Continuous	U/L
33	AST	Aspartate aminotransferase	Continuous	U/L
34	TP	Total protein	Continuous	g/L
35	ALB	Albumin	Continuous	g/L
36	GLB	Globulin	Continuous	g/L
37	A/G	Albumin-to-globulin ratio	Continuous	Ratio
38	TBil	Total bilirubin	Continuous	mol/L
39	DBil	Direct bilirubin	Continuous	mol/L
40	IBil	Indirect bilirubin	Continuous	mol/L
41	Glu	Glucose	Continuous	mmol/L
42	TC	Total cholesterol	Continuous	mmol/L
43	TG	Triglycerides	Continuous	mmol/L
44	HDL	High-density lipoprotein	Continuous	mmol/L
45	LDL	Low-density lipoprotein	Continuous	mmol/L
46	Urea	Urea	Continuous	mmol/L
47	Crea	Creatinine	Continuous	mol/L
48	UA	Uric acid	Continuous	mol/L
49	GFR	Glomerular filtration rate	Continuous	$mL/min/1.73m^2$
50	FOBT	Fecal occult blood test	Categorical	0 = Negative; 1 = Positive

Table S2. Performance of stacking ensemble models for MSI prediction. All results are reported on the independent test set. Best results for each metric are highlighted in bold.

Stacking Ensemble	Accuracy	Precision	Recall	AUROC
STK-1	**0.784**	**0.947**	**0.602**	**0.784**
STK-2	0.751	0.941	0.537	0.751
STK-3	0.753	0.941	0.540	0.753
STK-4	0.781	0.946	0.596	0.781
STK-5	0.777	0.945	0.588	0.777
STK-6	0.753	0.941	0.540	0.753
STK-7	0.754	0.946	0.540	0.754

Note: Ensemble configurations combine clinical and image-based models as follows: STK-1 = LR + GBC + ViT + VGG; STK-2 = LR + RF + ViT + VGG; STK-3 = LR + RF + ResNet + ViT + VGG; STK-4 = LR + GBC + ResNet + ViT + VGG; STK-5 = LR + GBC + RF + ResNet + ViT + VGG; STK-6 = LR + RF + ResNet + ViT + VGG + EfficientNet STK–7 = LR + RF + ResNet + ViT + VGG + EfficientNet + DenseNet.
